# Advances in the Surgical Treatment of Breast Cancer

**DOI:** 10.3390/curroncol30110693

**Published:** 2023-10-31

**Authors:** Rona Cheifetz, Elaine McKevitt

**Affiliations:** Department of Surgery, Faculty of Medicine, University of British Columbia, Vancouver, BC V5Z 1M9, Canada; emckevitt@providencehealth.bc.ca

Breast cancer is the most commonly occurring cancer in women and has become the most common cancer diagnosed worldwide [1]. In 2020 alone, 2.3 million women were diagnosed with breast cancer globally. Whereas multimodality therapy, including combinations of chemotherapy, hormonal blockade, immunotherapy, and surgery, is used for the management of breast cancer, surgery remains the mainstay of treatment and is a component of care of almost all patients diagnosed with nonmetastatic disease.

Yet, it has been more than three decades since Fisher [2] and Veronisi [3] published papers validating lumpectomy for the management of breast cancer, and more than two decades since Giuliano [4] and Veronisi [5] published their papers on sentinel lymph node biopsy for axillary staging. Surgical innovation tends to be a slower process than what is observed in other areas of cancer care. Nonetheless, innovation does occur, and the dissemination and adoption of new surgical approaches to the management of breast cancer are essential for optimizing patient outcomes.

This Special Issue focuses on the advances in the surgical management of breast cancer including expanding our understanding of when surgery should be employed in the diagnosis of breast cancer, understanding and minimizing the sequelae of surgical procedures, and considering treatment in the context of what is important to patients. 

Patients and clinicians remain concerned about making an accurate diagnosis of breast lesions and the possibility of missing breast cancer. Significant progress has been made over the past 30 years, with core needle biopsy replacing surgical excision for diagnosis in more than 95% of patients. The ongoing role of surgical excision in the context of expanding and improved breast imaging is a current focus of ongoing research. Park et al.^a^ assessed the role of vacuum-assisted breast biopsy alone in the diagnosis and management of breast cancer. Azam et al.^b^ reviewed mesenchymal tumors of the breast, which are a challenging group of lesions as they are infrequently encountered, and data to guide treatment are lacking.

Concern remains for both patients and providers regarding the sequelae of breast cancer surgical outcomes. The landmark trials establishing the safety of lumpectomy in the treatment of breast cancer were performed in the era before routine biomarker testing and systemic therapy in breast cancer. The role of mastectomy and lumpectomy and advances in techniques including reconstruction continue to be evaluated in the context of modern treatment. Greater understanding of patient perspectives, individual cancer, psychosocial, and medical risk factors, and surgical complications has helped to define the roles of different surgical approaches. Augustin et al.^c^ assessed donor site morbidity and quality of life after autologous flap reconstruction. McKevitt et al.^d^ compared preoperative health and patient-reported outcomes for different breast cancer procedures. Nair et al.^e^ reviewed the evidence for breast reconstruction in the setting of inflammatory breast cancer, and Schick et al.^f^ reviewed the role of the surgeon in germline testing for newly diagnosed breast cancer patients. All these articles focus on optimizing the selection of a surgical approach for patients.

Surgical management of breast cancer has traditionally involved surgery on the breast and axillary lymph nodes. One of the feared complications of axillary surgery is lymphedema, and this has driven innovation to minimize this complication. Sentinel lymph node biopsy has replaced axillary dissection for the staging of patients with clinically node-negative disease, and increasing the role of sentinel node biopsy has been the focus of many studies since. Lovrics et al.^g^ reviewed the evidence for performing sentinel node biopsy in patients presenting with positive nodes who are treated with surgery initially. Lustig et al.^h^ and Deban et al.^i^ looked at immediate lymphatic reconstruction for the prevention of lymphedema for patients that still require axillary lymph node dissection.

The ongoing improvement in the outcomes for breast cancer patients is exciting and has been driven by continuous innovation and research. Now, most women are expected to survive their cancer, although we are seeing increasing concerns about the sequelae of treatment. Currently, a main focus of research is in de-escalating treatment in order to optimize survival outcomes and decrease complications. Both the original research in this Special Issue and the reviews raise the importance of ongoing data collection and evaluation of our treatment paradigm and raise awareness of important areas that are still not completely understood. We hope that the articles in this Special Issue will help to refine the roles of surgical procedures in the diagnosis and treatment of breast cancer and will inspire clinicians and researchers to continue to work to improve breast cancer surgery.

## List of Contributions

a. Park, J.H.; Ahn, S.E.; Kim, S.; Kwon, M.J.; Suh, Y.J.; Kim, D. Complete Surgical Excision Is Necessary following Vacuum-Assisted Biopsy for Breast Cancer. *Curr. Oncol.* **2022**, *29*, 9357–9364. https://doi.org/10.3390/curroncol29120734.
b. Azam, R.; Mrkonjic, M.; Gupta, A.; Gladdy, R.; Covelli, A.M. Mesenchymal Tumors of the Breast: Fibroblastic/Myofibroblastic Lesions and Other Lesions. *Curr. Oncol.* **2023**, *30*, 4437-4482. https://doi.org/10.3390/curroncol30050338.
c. Augustin, A.; Pülzl, P.; Morandi, E.M.; Winkelmann, S.; Schoberleitner, I.; Brunner, C.; Ritter, M.; Bauer, T.; Wachter, T.; Wolfram, D. Donor-Site Morbidity and Quality of Life after Autologous Breast Reconstruction with PAP versus TMG Flap. *Curr. Oncol.* **2022**, *29*, 5682–5697. https://doi.org/10.3390/curroncol29080448.
d. McKevitt, E.; Saleeb, M.; Liu, G.; Warburton, R.; Pao, J.-S.; Dingee, C.; Bazzarelli, A.; Tang, K.; Crump, T.; Sutherland, J.M. Differences in Preoperative Health-Related Quality of Life between Women Receiving Mastectomy or Breast Conserving Surgery in a Prospectively Recruited Cohort of Breast Cancer Patients. *Curr. Oncol.* **2023**, *30*, 118–129. https://doi.org/10.3390/curroncol30010010.
e. Nair, A.G.; Ko, G.T.Y.; Semple, J.L.; Lim, D.W. Breast Reconstruction Use and Impact on Surgical and Oncologic Outcomes Amongst Inflammatory Breast Cancer Patients—A Systematic Review. *Curr. Oncol.* **2023**, *30*, 6666–6681. https://doi.org/10.3390/curroncol30070489.
f. Schick, S.; Manghelli, J.; Ludwig, K.K. The Role of the Surgeon in the Germline Testing of the Newly Diagnosed Breast Cancer Patient. *Curr. Oncol.* **2023**, *30*, 4677–4687. https://doi.org/10.3390/curroncol30050353.
g. Lovrics, O.; Tao, B.; Parvez, E. Safety and Accuracy of Sentinel Lymph Node Biopsy Alone in Clinically Node-Positive Patients Undergoing Upfront Surgery for Invasive Breast Cancer: A Systematic Review. *Curr. Oncol.* **2023**, *30*, 3102–3110. https://doi.org/10.3390/curroncol30030235.
h. Lustig, D.B.; Temple-Oberle, C.; Bouchard-Fortier, A.; Quan, M.L. Incorporating Lymphovenous Anastomosis in Clinically Node-Positive Women Receiving Neoadjuvant Chemotherapy: A Shared Decision-Making Model and Nuanced Approached to the Axilla. *Curr. Oncol.* **2023**, *30*, 4041–4051. https://doi.org/10.3390/curroncol30040306.
i. Deban, M.; McKinnon, J.G.; Temple-Oberle, C. Mitigating Breast-Cancer-Related Lymphedema—A Calgary Program for Immediate Lymphatic Reconstruction (ILR). *Curr. Oncol.* **2023**, *30*, 1546–1559. https://doi.org/10.3390/curroncol30020119.
